# HCV monoinfection and HIV/HCV coinfection enhance T-cell immune senescence in injecting drug users early during infection

**DOI:** 10.1186/s12979-016-0065-0

**Published:** 2016-03-31

**Authors:** Bart P. X. Grady, Nening M. Nanlohy, Debbie van Baarle

**Affiliations:** Department of Research, Cluster Infectious Diseases, Public Health Service, Amsterdam, The Netherlands; Center for Infection and Immunity Amsterdam (CINIMA), Academic Medical Center, Amsterdam, The Netherlands; Department of Immunology, University Medical Center Utrecht, Utrecht, The Netherlands; Department of Internal Medicine, University Medical Center Utrecht, Utrecht, The Netherlands; Present address: Department of Immune Mechanisms, Center for Infectious Disease Control, National Institute for Public Health and the Environment (RIVM), Bilthoven, The Netherlands

**Keywords:** Substance abuse, People who inject drugs, Frailty, Immunosenescence, Longitudinal

## Abstract

**Background:**

Injecting drug users (IDU) are at premature risk of developing multimorbidity and mortality from causes commonly observed in the elderly. Ageing of the immune system (immune-senescence) can lead to premature morbidity and mortality and can be accelerated by chronic viral infections. Here we investigated the impact of HCV monoinfection and HIV/HCV coinfection on immune parameters in (ex-) IDU. We analyzed telomere length and expression of activation, differentiation and exhaustion markers on T cells *at baseline (t = 1) and at follow-up (t = 2)* (median interval 16.9 years) in IDU who were: HCV mono-infected (*n* = 21); HIV/HCV coinfected (*n* = 23) or multiple exposed but uninfected (MEU) (*n* = 8).

**Results:**

The median time interval between t = 1 and t = 2 was 16.9 years. Telomere length within CD4^+^ and CD8^+^ T cells decreased significantly over time in all IDU groups (*p* ≤ 0.012). CD4^+^ T-cell telomere length in HCV mono-infected IDU was significantly reduced compared to healthy donors at t = 1 (*p* < 0.008). HIV/HCV coinfected IDU had reduced CD4^+^ and CD8^+^ T-cell telomere lengths (*p* ≤ 0.002) to healthy donors *i* at t = 1. This was related to persistent levels of immune activation but not due to increased differentiation of T cells over time. Telomere length decrease was observed within all T-cell subsets, but mainly found in immature T cells (CD27^+^CD57^+^) (*p* ≤ 0.015).

**Conclusions:**

HCV mono-infection and HIV/HCV coinfection enhance T-cell immune-senescence. Our data suggest that this occurred early during infection, which warrants early treatment for both HCV and HIV to reduce immune senescence in later life.

**Electronic supplementary material:**

The online version of this article (doi:10.1186/s12979-016-0065-0) contains supplementary material, which is available to authorized users.

## Background

As people age, the immune system exhibits age-associated changes resulting in impaired immunity. This so-called immune senescence is a complex multifactorial phenomenon characterized by a number of features including: i) reduced number of naïve T cells; ii) increased frequencies of differentiated CD28^-^CD57^+^ T cells that have a reduced proliferative capacity; iii) reduced CD4/CD8 ratio; oligoclonal expansion of CD8 T cells, and iv) progressive shortening of telomeres [1–3]. Telomeres are repetitive (TTAGGG)_n_ nucleotide sequences that shorten with each cell division [4]. Among people aged over 60 years, short leukocyte telomere length has been associated with higher mortality rates from infectious diseases [5].

People who inject drugs (injecting drug users, IDU) are at increased risk of contracting both acute and chronic infections [6, 7]. The prevalence of HCV antibodies in IDU ranges from 15–98 % [8, 9]. Upon HCV infection, 75 % of individuals progress to chronic infection and are at risk for progressive liver disease, liver cirrhosis and hepatocellular carcinoma [10]. The worldwide prevalence of HIV infection among IDU is estimated to be 18 % [11]. With the advent of combination antiretroviral therapy (cART) and decline in drug-related causes of death, the mean age of IDU is increasing [12, 13] and IDU are at premature risk of developing multimorbidity and mortality from causes commonly observed in the elderly [14, 15].

Immunological changes and increased levels of inflammation could form the basis of this premature burden of morbidity and mortality among ageing DU. Progression of immune senescence was shown to be accelerated by chronic viral infections such as HIV through (long-term) continuous immune activation [16, 17]. Despite adequate combination antiretroviral therapy (cART), HIV infected individuals have increased risk for non-AIDS morbidity as compared to age-matched controls [18, 19]. There is a growing body of literature that suggests that HCV has a role in extrahepatic morbidity and mortality likely through a similar mechanism of immune activation [20, 21]. Indeed, like HIV, HCV infection also leads to PD-1^high^ and TIM-3^high^ T cells, a phenotype associated with exhaustion due to persistent antigenic pressure [22]. In addition to HIV and HCV monoinfection, HIV/HCV coinfected individuals do not only seem to have increased risk for liver disease progression [23] but also progression to AIDS [24], which suggests that both viruses could enhance each other’s disease progression [25].

To assess the impact of an infection with HCV and HIV/HCV specifically, we studied parameters associated with immune senescence. To this end, we included IDU with HCV mono- or HIV/HCV coinfection. As a control group to control for use of cocaine, opioid and social practices connected with drug use, we studied IDU with similar injecting risk behavior that where multiple exposed but uninfected (MEU) from the Amsterdam Cohort Studies (ACS) among drug users, at two time-points during follow-up >15 years apart. To address the severity of immune senescence parameters, we compared these between the specific IDU groups and healthy individuals.

## Results

### Study population

We included 23 HIV/HCV coinfected, 21 HCV infected and 8 MEU DU (Table 1) who all injected drugs for at least 2 years. The number of years of injecting risk behavior was comparable between groups, although MEU IDU reported less injecting in the past 6 months prior to the baseline time point (*p* = 0.07). At baseline, 1 out of 23 (4.5 %) HIV/HCV IDU was on combination antiretroviral therapy (cART) and this number increased to 20 out of 23 (87.0 %) at follow-up. The remaining three HIV/HCV cases never received cART. For those who received cART the median time since start cART was 7.1 years (IQR 2.1–10.7). Median nadir CD4 count was 130 cells/mm^3^ (IQR 90–210).

**Table 1:** Baseline and follow-up characteristics of the study population

	HD#	MEU	HCV	HIV/HCV	*P*-value
Number	22	8	21	23	
General characteristics					
Gender, n male (%)	*	7 (87.5)	15 (71.4)	14 (60.9)	0.36
Western ethnicity, n (%)	*	8 (100.0)	21 (100.0)	19 (82.6)	0.13
Ever injected drugs, n (%)	*	8 (100.0)	21 (100.0)	23 (100.0)	1.00
Years of injecting (IQR)	*	6.7 (6.1–13.6)	13.4 (5.4–19.6)	9.0 (6.4–14.6)	0.20
Baseline (T = 1)					
Age, median (IQR)	36.4 (31.5–40.1)	32.8 (28.7–35.2)	34.4 (30.7–37.5)	35.2 (32.6–39.8)	0.30
Sample since study entry	*	12.7 (0–47.6)	12.6 (1.7–31.7)	14.7 (0–25.8)	0.98
(months), median (IQR)					
Year of sample,	*	1992 (1989–1994)	1992 (1990–1994)	1991 (1989–1993)	0.43
median (IQR)					
Injecting past 6 months(%)	*	1 (12.5)	14 (66.7)	16 (73.9)	0.07
CD 4 cell counts 10^6^ cells/L,	*	*	*	590 (470–742)	*
median (IQR)					
cART, n (%)	*	*	*	1 (4.5)	*
Follow-up (T = 2)					
Age, median (IQR)	52.7 (48.3–57.6)	51.7 (49.2–54.8)	51.7 (47.4–55.5)	50.4 (47.7–54.2)	0.97
Injecting past 6 months (%)	*	1 (12.5)	4 (19.0)	4 (17.9)	0.92
CD 4 cell counts 10^6^ cells/L,	*	*	*	341 (233–663)	*
median (IQR)					
cART, n (%)	*	*	*	20 (87.0)	*
Years on cART, median (IQR)	*	*	*	7.1 (2.1–10.7)	*

*cART* combination anti-retroviral therapy, *HCV* Hepatitis C virus, *HD* Healthy donor, *IQR* Interquartile range, *MEU* Multiple exposed but uninfected with HCV or HIV

#HD *at T = 1 and T = 2* are not the same individuals; * Data unavailable

### Flowcytometric analyses of telomere length

Using flow-FISH, telomere length can be measured in distinct cell populations without prior cell sorts [26]. Here we extended the flow-FISH protocol [27, 28] to a 5 color- flow-FISH (incorporating the phenotypic markers CD3, CD8, CD27 and CD57) enabling us to investigate CD4 and CD8 phenotypic T cell subsets in one sample. The assay has been shown to be sensitive enough to detect significant decreases in telomere length [28].

**Fig. 1:**
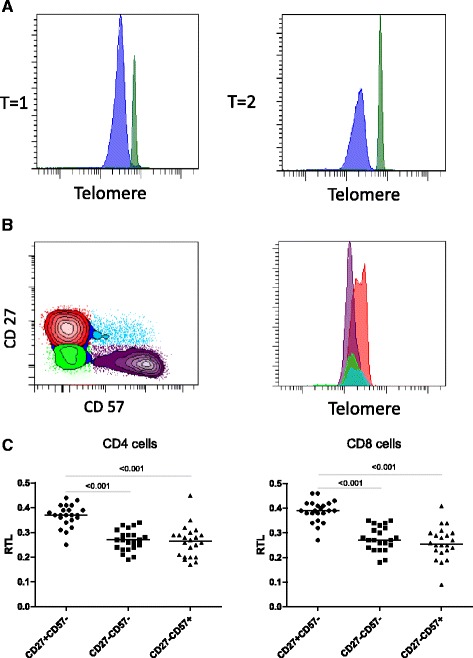
Flowcytometric analysis of telomere length within T-cell subsets. **a** Representative histograms of CD8+ telomere length (blue) and calf thymocytes (green) at the baseline timepoint (t = 1) and a follow-up timepoint more than 15 years later (t = 2). **b** Telomere length analysis (or relative Telomere lengths) within T-cell subsets defined by CD27 and CD57-expression (*left panel*) as CD27 ^+^ CD57^-^ (red), CD27^-^CD57^-^ (green) and CD27^-^CD57^+^ (purple). **c** Differences in relative telomere length (RTL) in healthy donors between immature (CD27 ^+^ CD57^-^), mature (CD27^-^CD57^-^) and mature differentiated (CD27^-^CD57^+^) T cells stratified for CD4 (*left panel*) and CD8 T cells (*right panel*). The black lines represent median values. Statistical analyses were performed using Kruskal-Wallis and post hoc Mann-Whitney *U* test, a two sided *p*-value <0.05 was considered statistically significant

During ageing the relative telomere length (RTL) decreases, as shown in Fig. 1a in CD8^+^ T cells over a period of 17 years. Using CD27/CD57 expression for defining immature (CD27^+^CD57^-^), mature (CD27^-^CD57^-^) and mature differentiated (CD27^-^CD57^+^) phenotypes [29, 30] (Fig. 1b), we were also able to show differences in telomere length between these subsets (Fig. 1c). In both CD4^+^ and CD8^+^ T cells, immature cells had significantly longer RTL compared to mature and mature differentiated cells (*p* < 0.001). Shortened telomeres have been associated with CD57 expression on the surface of T-cells [31]. Here we show that loss of CD27 expression is already associated with reduced RTL in CD4^+^ and CD8^+^ T cells. Mature and mature differentiated cells have similar RTL, indicating that they have undergone comparable rounds of proliferation (Fig. 1c).

### Telomere length decreases over time in CD4^+^ and CD8^+^ T cells and is mostly affected by HIV/HCV coinfection

**Fig. 2:**
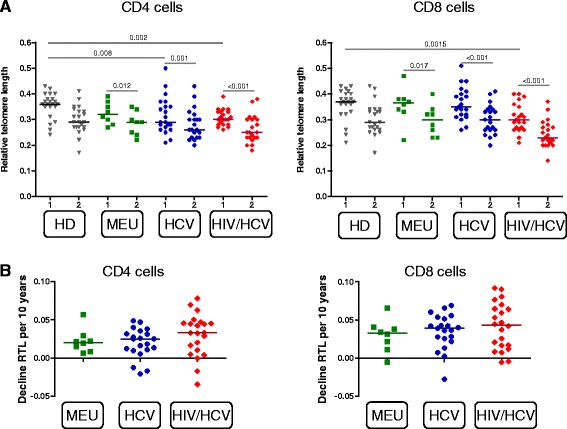
Telomere length decreases over time in CD4^+^ and CD8^+^ T cells. **a** RTL of peripheral CD4 T cells (*left panel*) and CD8 T cells (*right panel*) over time of: healthy donors (HD); multiple exposed uninfected (MEU) injecting drug users (IDU); HCV monoinfected IDU and HIV/HCV coinfected DU. RTL was measured in the first available sample since study entry (t = 1) and the most recent sample (t = 2) of MEU, HCV monoinfected and HIV/HCV coinfected DU. HD at time point 1 and 2 are not the same individuals. The median time interval for all groups between time point 1 and 2 was 16.9 years. Medians are depicted in the scatterplots. Wilcoxon-signed rank test was used for comparison within groups with the same individuals (MEU, HCV and HIV/HCV). Kruskall-Wallis test were used to compare between groups followed by post hoc Mann-Withney U tests. **b** Median levels of RTL decrease over time calculated per individual per 10 years for CD4 T cells (*left panel*) and CD8 T cells (*right panel*)

We investigated whether there was a decrease in RTL among CD4^+^ and CD8^+^ T cells over time (Fig. 2a). The RTL of CD4^+^ T cells decreased significantly over time in all IDU groups (*p* ≤ 0.012). An impact at baseline of HCV monoinfection and HIV/HCV coinfection was observed among the RTL in CD4^+^ T cells compared to healthy donors (*p* = 0.008 and *p* = 0.002 respectively). Among CD8^+^ T cells the RTL also decreased in all IDU groups (*p* ≤ 0.017). The median RTL of CD8^+^ T cells from HIV/HCV coinfected IDU at baseline was significantly lower than in healthy donors (*p* = 0.0015) and comparable to the median RTL of healthy donors, HCV and MEU at follow-up (T = 2). In a sensitivity analysis, using a linear regression model with age included as a fixed variable, we demonstrated that the observed difference as mentioned above were independent of age (Additional file 1: Table S1). To analyse the decline in RTL per individual, the 10 year RTL decline was calculated. With increasing age, the RTL decline rate did not statistically differ between the study groups (Fig. 2b). Taken together, these results suggest The effect of these infections occurred before the first time point of the study of HIV/HCV coinfection on immune senescence.

### Lower telomere lengths in immature T cells in HIV/HCV coinfected IDU coincides with increased numbers of differentiated cells

**Fig. 3:**
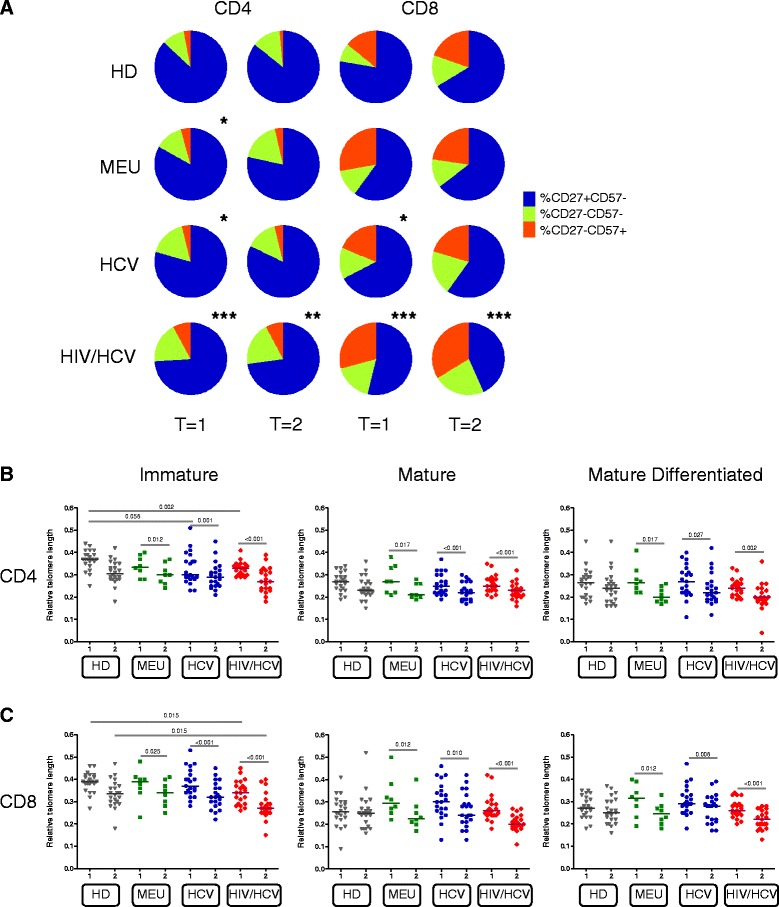
No enhanced T-cell differentiation in time and lower relative telomere lengths (RTL) in immature CD4^+^ and CD8^+^ T cells. **a** Pie charts of normalised median frequencies of immature (blue), mature (green) and mature differentiated (red) CD4^+^ (*left panels*) and CD8^+^ T cells (*right panels*). Frequencies of immature cells were compared with HD for CD4^+^ and CD8^+^ T cells for time-point 1 and for time-point 2. *P*-values were calculated using the Mann- Whitney *U* test. *p < 0.05; **p < 0.01; *** < 0.001. **b** Relative telomere length (RTL) of peripheral CD4 T cell subsets (**b**) and CD8 T cell subsets (**c**) of: healthy donors (HD); multiple exposed uninfected (MEU) drug users (DU); HCV monoinfected IDU and HIV/HCV coinfected DU. RTL was measured in the first available sample since study entry (t = 1) and the most recent sample (t = 2) of MEU, HCV monoinfected and HIV/HCV coinfected DU. Subsets are depicted as follows: immature (CD27^+^CD57^-^), mature (CD27^-^CD57^-^) and mature differentiated (CD27^-^CD57^+^). HD at time point 1 and 2 are not the same individuals. The median time interval for all groups between time point 1 and 2 was 16.9 years. Medians are depicted in the plots. Wilcoxon-signed rank test was used for comparison within groups with the same individuals (MEU, HCV and HIV/HCV). Kruskall-Wallis test were used to compare between groups followed by post hoc Mann-Withney U tests

Persistent antigenic stimulation leads to linear differentiation of naïve cells losing CD27 [32, 33] and gradually gaining CD57 [30], resulting in a decreased capacity to proliferate [34]. Therefore long-term effects of persistent antigenic stimulation could be reflected in the percentage of immature, mature and mature differentiated T-cell subsets. As shown in Fig. 3a the proportion of immature CD4^+^ and CD8^+^ T cells was significantly lower among HIV/HCV coinfected IDU than healthy donors at both baseline and follow-upt (*p* < 0.01) (Fig. 3c) fitting with the lower telomere lengths in this patient group. However, we did not observe a significant increase in the percentage of differentiation over time within each of the study groups, indicating that loss of telomere length over time is not simply due to increased T-cell differentiation. Even more, the RTL significantly decreased over time in all T-cell subsets for all IDU groups (≤0.027, Fig. 3b and c). In addition, in immature CD8^+^ T cells, the RTL in HIV/HCV infected IDU was significantly lower compared to healthy donors (*p* = 0.015). The CD27^+^CD57^-^ immature CD4^+^ T cells from young IDU with HCV or HIV/HCV also had a lower RTL than healthy donors (*p* = 0.056 and *p* < 0.001 respectively). Thus, the decrease in telomere length over time does not seem to be due to enhanced differentiation of T cells, but affects all T-cell subsets.

### Increased levels of activation and exhaustion in peripheral T cells of HCV monoinfected and HIV/HCV coinfected DU

**Fig. 4:**
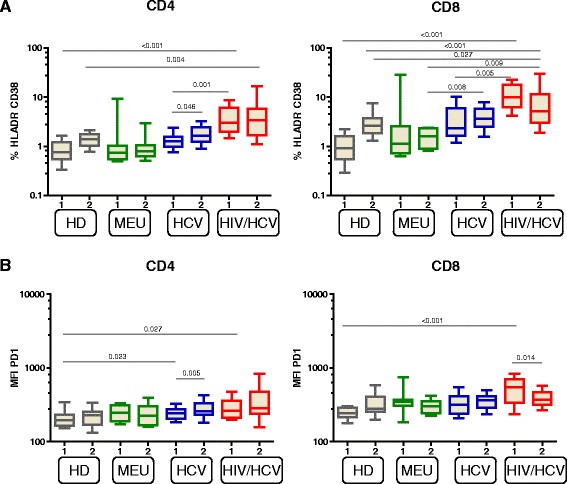
Levels of immune activation and exhaustion are increased in HCV/HIV coinfected injecting drug users (IDU). Percentages of HLA-DR/CD38 positive peripheral CD4^+^ T cells and CD8^+^ T cells (**a**) of: healthy donors (HD); MEU IDU; HCV monoinfected IDU and HIV/HCV coinfected DU. RTL was measured in the first available sample since study entry (t = 2) and the most recent sample (t = 2) of MEU, HCV monoinfected and HIV/HCV coinfected DU. **b** Median fluorescent intensity (MFI) of PD1 in peripheral CD4^+^ T cells and CD8^+^ T cells. Box and whisker plots show the median and 10–90 percentiles. The Wilcoxon-signed rank test was used for comparison within groups with the same individuals (MEU, HCV and HIV/HCV). Kruskall-Wallis test were used to compare between groups followed by post hoc Mann-Withney U tests

To investigate whether the observed decrease in RTL over time could be due to enhanced immune activation, we analyzed the expression of HLA-DR and CD38 on T cells. IDU with HIV/HCV coinfection had a significantly higher frequency of CD4^+^ and CD8^+^ T-cell activation (HLADR^+^CD38^+^) compared to healthy donors at both baseline and follow-up (*p* < 0.004, Fig. 4). IDU with HCV monoinfection had higher levels of CD8^+^ T cell activation at baseline compared to healthy donors (*p* < 0.001), but this effect diminished over time. The level of CD4^+^ and CD8^+^ T cell activation declined over time in HIV/HCV infected DU, but was still higher than in healthy donors (*p* < 0.001). The expression of activation markers was also significantly higher in HCV and HIV/HCV infected IDU compared to MEU DU. Interestingly, young MEU IDU were comparable to young healthy donors with respect to immune activation, which suggests there was no impact of drug use or social practices on immune activation. However, the levels of CD38 and HLA-DR among MEU IDU remained stable over time, suggesting that MEU IDU may actively suppress immune activation.

Persistent antigen exposure does not only lead to a rapid turnover and telomere erosion but can also lead to a subset of T cells that become functionally exhausted. To investigate whether T-cell exhaustion is upregulated by HIV and/or HCV we evaluated programmed death factor 1 (PD-1) expression levels, shown to be marker of exhaustion in chronic viral diseases but increasingly also considered as activation marker after acute infection (to control T-cell activity). At baseline both CD4^+^ and CD8^+^ T cells of HIV/HCV infected IDU expressed higher levels of PD-1 than healthy donors. Over time, the CD8 PD-1 expression of HIV/HCV infected IDU declined significantly (*p* = 0.014) to a level comparable to healthy donors, most likely due to cART. Among HCV monoinfected IDU the expression of PD-1 in CD4^+^ T cells was higher compared to healthy donors (*p* = 0.023). Even though PD-1 expression in these cells significantly increased over time (*p* = 0.005) the expression level was comparable to older healthy donors, MEU and HIV/HCV coinfected DU. Thus HIV/HCV coinfection leads to both general increased immune activation and increased PD-1 expression.

## Methods

### Study population

Study subjects were recruited from the ACS among DU, an open, prospective cohort study to investigate the prevalence, incidence, and risk factors of HIV infections and other blood-borne diseases [35]. Enrollment is voluntary, anonymous, and written informed consent is obtained from each participant at the intake visit. The medical ethics committee of the Academic Medical Center approved this observational study. Blood is drawn each visit for laboratory testing and storage of peripheral blood mononuclear cells (PBMC) and serum. HIV testing and HCV testing have been described before [36]. In short, all participants were prospectively tested for HIV antibodies and were confirmed by Western blot. Chronic HCV infection was defined by the presence of positive anti-HCV tests and the presence of HCV RNA at multiple time-points during follow up, without evidence for spontaneous clearance of HCV. None of HCV-infected participants received HCV-treatment.

For this study we included three groups of DU, namely: IDU who had an HIV/HCV coinfection (*n* = 23), IDU who had a chronic HCV infection (*n* = 21) and as a control for a drug using career IDU who were multiple exposed but uninfected (MEU) (*n* = 8) (Table 1). Subjects were included if they had an injecting drug use career greater than 2 years, were aged between 43 and 60 years and had PBMCs available. In addition to these follow-up samples we also included the first available PBMCs sample since study entry in the ACS for each subject. Unfortunately we were unable to include healthy donors with stored PBMC over the same time period. Therefore, to compare the study groups to healthy donors we recruited 2 groups of anonymous healthy donors from the blood bank, one aged between 43–60 years and one aged between 23–43 years, in order to match the ages of our study groups. In order to donate blood, voluntary participating individuals are tested for HIV, HBV, HCV and HEV. The blood bank actively screens for IDU and men who have sex with men or a history of IDU or men who have sex with men. These individuals were excluded from blood donation.

## PBMC storage

From all study participants, PBMCs were isolated from heparinized blood using a Ficoll-Hypaque density gradient centrifugation and cryopreserved using a computerized freezing system in liquid nitrogen within 24 h of collection.

## Flow cytometric analyses

Stored PBMCs were rapidly thawed and 1*10^6^ cells were stained in PBS with 0.5 % bovine serum albumin (BSA) and 0.1 % sodium azide using combinations of the following antibodies: CD4 Pacific Blue, CD3 AlexaFluor700, HLA-DR PerCP (Biolegend), CD8 Horizon V500, CD27 APC-eFluor780 (eBioscience), CD38 PE (Caltag) and PD-1 PerCP-Cy5.5. Cells were incubated with the antibodies for 20 min at 4 °C. After washing with PBS/0.5 % BSA, cells were fixed with Cellfix (BD) and directly analyzed by flow cytometry. For each sample a minimum of 100,000 cells were acquired using a LSRII FACS (BD) and data were processed using FACSDiva 6.0 software (BD).

## Flowcytometric analysis of telomere length in T cell subsets

Telomere length of PBMCs was assessed using a five color flow cytometry fluorescent in situ hybridization (flow-FISH) protocol, adapted from Baerlocher et al. [27] Here, telomeres are hybridized to an AlexaFluor488 labeled peptide nucleic acid (PNA) telomeric (C3TA2)^3^ probe and subsequently analyzed by flow cytometry. In short, stored PBMCs were rapidly thawed and 2*10^6^ cells were stained with heat-stable fluorochrome-labeled antibodies for CD3 Pacific Blue (eBioscience), CD8 V500 (BD), CD27 Alexa fluor 647 (BD) and CD57-biotin (Biolegend), followed by streptavidin-Cy3 (Sigma). After washing, the cells were fixed with bis(sulfosuccinimidyl)suberate (BS^3^, Pierce) for 30 min at 4 °C in the dark. Cells were washed with PBS and incubated for 10 min with an hybridization solution, with and without the PNA probe and 15 min at 82 °C to denature the DNA. After 1 h of hybridization at room temperature and in the dark, cells were washed and analyzed immediately by flow cytometry. Samples were gated on live, singlet CD3^+^ T cells. Calf thymocytes were included in each experiment as an internal control. The gating strategy is shown in Additional file 2: Figure S1. Relative telomere length (RTL) of each sample was calculated as the ratio between the median fluorescent intensity (MFI) of the T cell subset of interest with probe (minus the MFI without probe) divided by the MFI of the calf thymocytes with probe (minus the MFI without probe). All experiments were performed in duplo and RTLs were averaged per sample.

## Statistical analyses

To test for statistical significance between groups we used the Kruskall-Wallis test and if significant followed by post-hoc Mann-Whitney *U* test. Comparisons within groups (related samples) were made using the paired Wilcoxon signed rank test, otherwise the Mann-Whitney *U* test was used. A two sided *p*-value <0.05 was considered statistically significant. To investigate whether the decline in RTL could be confounded by age we performed a sensitivity analysis using a linear regression model with age as a fixed variable. All analyses were performed using SPSS (version 20.0; SPSS Inc.) statistical software. Graphs were made using Graphpad (version 6.1; GraphPad Software, Inc.)

## Discussion

In this longitudinal study we observed significantly decreased telomere lengths among ageing HIV/HCV coinfected IDU as compared to healthy donors. In the period in which IDU had no access to cART, the impact of HIV/HCV on telomere length was noticeable already at the first timepoint in infection that we analysed, in both the CD4 and CD8 T-cell compartment with significantly reduced telomere lengths. During a period of 16 years we observed no increased decline of telomere length between the study groups. These data suggest that the lower telomere lengths were induced earlier in infection. HCV monoinfected IDU had significantly decreased telomere lengths in their CD4^+^ T cells, but CD8^+^ T cells were not affected by increased telomere erosion. Over time we observed no increase in the percentage of differentiated cells in each study group, but we did observe a continued decline of telomere erosion. Therefore it is unlikely that T-cell differentiation alone explains the continued telomere erosion. Telomere decline could be explained by increased peripheral levels of activation (HLA-DR^+^CD38^+^), mature differentiated (CD27^-^CD57^+^) cells and exhaustion (PD-1) in peripheral T cells of HCV monoinfected and HIV/HCV coinfected IDU which indicates a state of chronic immune activation.

As expected, we observed that telomere length decreased over time in all IDU groups. However this was independent of viral coinfections (HCV or HIV/HCV). Interestingly, at a relatively young age the telomere length of predominantly CD8^+^ T cells, but also CD4^+^ T cells, was markedly decreased in HIV/HCV coinfected individuals and was comparable to more than 15 year older healthy donors. As most HIV/HCV coinfected individuals were cART naïve early during infection, the immune system responds to HIV with high levels of activation and proliferation rates [37]. Consequently HIV drives T cells to increasingly differentiated phenotypes that are oligoclonally expanded, less functional and more prone to apoptosis [38]. We demonstrated that loss of telomere length is not simply due to increased differentiation but mainly to continued immune activation. Importantly, this study demonstrates that the loss in telomere length mainly occurred at the first time-point in infection that we analysed and was not restored to the level of healthy individuals with the initiation of cART. We could not rule out that cART, via telomerase inhibition [39], negatively affects telomere length. However a recent cross-sectional study by Zanet et al. demonstrated no association between low telomere length and cART exposure [40].

Here we found that HCV monoinfected IDU had lower CD4^+^ T cell telomere lengths than healthy donors at the first timepoint in infection that we analysed, suggesting that HCV on its own may have an effect on immune senescence. However, CD8^+^ T cell telomere length was not affected. Unfortunately we had no clinical outcomes to relate to, but a hospital-based study found that, independent of age, decreased CD4^+^ memory telomere length was associated with increased liver fibrosis [41]. In addition, longer CD4^+^ and CD8^+^ T cell telomere lengths were both associated with a sustained virological response following HCV treatment. We demonstrated that in HCV monoinfected IDU the decreased telomere length in CD4^+^ T cells occurred mainly in the immature T cells. Although this population consists of both naïve and central memory cells [42], reduced numbers of CD4 naïve T cells and reduced recent thymic emigrants have been associated with HCV infection, especially if fibrosis is present [43, 44]. This fits with a model in which CD4^+^ T cells are continuously activated during persistent HCV infection, especially when the infection aggravates.. However, due to a lack of samples we were unable to investigate the specific responses of HIV/HCV coinfected DU.

The exact mechanisms through which HIV, HCV and natural ageing collectively affect disease progression remains to be resolved. Accumulating evidence points towards a role for systemic immune senescence affecting multiple organs/tissues. Data from a recent study among IDU demonstrated that higher levels of interleukin 6, a proinflammmatory cytokine, were independently associated with HCV monoinfection, HIV/HCV coinfection and increasing age [45]. Decreased telomere length has also been associated with atherosclerosis and cardiovascular disease, and is likely to be correlated with interleukin 6 levels [46].

Of interest, MEU IDU tended to have lower levels of immune activation compared to healthy donors. This special group of IDU has been shown to have detectable HIV-specific [47] and HCV-specific T-cell responses [48], indicating their exposure to both infections. The notion of a naturally occurring resistance to certain viral pathogens has major implications for T-cell vaccine development. In a recent study though, robust activation of natural killer cells, but not HCV-specific adaptive immune responses, was associated with protection against infection with HCV among MEU DU [49].

There were several limitations in this study. Due to instability to heat we were unable to use CD45RA and CCR7 as markers of memory and differentiation in our assay. Interestingly, it did enable us to demonstrate that loss of CD27 was significantly associated with telomere loss in both CD4^+^ and CD8^+^ T cell, which occurred before the upregulation of CD57 [31].

This study is limited by the unknown duration of HIV and HCV infection. However, as the observed peak incidence of HIV in Amsterdam occurred during the 80’s [50] we assumed that our first time point of analysis was close to the actual infection time point. For HCV the observed peak prevalence also occurred during the 80’s. We demonstrated that the reduction in telomere length already occurred at the first time-point and that we did not find any difference in the rate of telomere length decline over a period of almost 17 years between MEU, HCV monoinfected and HIV coinfected IDU. This suggests that the telomere decline occurred earlier during infection. But, we can not rule out that the HIV or HCV infected IDU had lower telomere lengths pre-acquisition of HIV or HCV. To prove our hypothesis it would be of future interest to investigate telomere decline in HIV and HCV seroconverters. Unfortunately we had no access to bloodsamples of healthy donors followed over time. Because we used different healthy donors for the two time-points the decline in RTL could be biased by inter-individual variations.

## Conclusions

We found increased levels of immune senescence at the first timepoint that we analysed in HCV mono- and HIV/HCV coinfected DU. This suggests that HCV mono-infection and HIV/HCV coinfection enhance T-cell immune-senescence probably early during infection. As both viruses have detrimental long-term effects on morbidity and mortality, these data express the need for early treatment, both for HCV and HIV infection.

## Additional files

Additional file 1: Linear regression models of relative telomere length decline. (DOCX 14 kb) Additional file 2: Flowcytometric analysis of telomere length. Example of flowcytometric analysis of telomere length by flow-FISH, where calf thymocytes (red) can be distinguished from lymphocytes (blue), not only by forward and sideward scatter (left panel) but also by the lack of CD3 expression (right panel) (A&B). The cells were either hybridized to the peptide nucleic acid (PNA) probe (D) or underwent the same experimental conditions without the PNA probe (c) to account for the level of autofluorescence. (PDF 216 kb)
