# Supplementary material for: HCV monoinfection and HIV/HCV coinfection enhance T-cell immune senescence in injecting drug users early during infection
Source: Immun Ageing. 2016 Mar 31;13:10. doi: 10.1186/s12979-016-0065-0 (PMC4815107; doi:10.1186/s12979-016-0065-0)
Supplement: Additional file 1: — Linear regression models of relative telomere length decline. (DOCX 14 kb) [file 12979_2016_65_MOESM1_ESM.docx]

Supplementary table.

Linear regression models of relative telomere length decline.

|  | **CD 4** | | | **CD8** | | |
| --- | --- | --- | --- | --- | --- | --- |
| **T=1** | **β** | **SE** | **p** | **β** | **SE** | **p** |
| Group |  |  | 0.012 |  |  | 0.057 |
| HCV | -0.039 | 0.017 | 0.023 | 0.004 | 0.017 | 0.826 |
| MEU | -0.035 | 0.018 | 0.052 | -0.008 | 0.025 | 0.760 |
| HIV/HCV | -0.037 | 0.012 | 0.003 | -0.041 | 0.018 | 0.020 |
| HD | Ref |  |  | Ref |  |  |
| Age | -0.003 | 0.001 | 0.006 | -0.03 | 0.001 | 0.034 |
| **T=2** |  | | | | | |
| Group |  |  | 0.186 |  |  | 0.005 |
| HCV | -0.017 | 0.017 | 0.309 | -0.002 | 0.018 | 0.910 |
| MEU | -0.016 | 0.019 | 0.402 | -0.006 | 0.024 | 0.809 |
| HIV/HCV | -0.034 | 0.016 | 0.029 | -0.054 | 0.018 | 0.003 |
| HD | Ref |  |  | Ref |  |  |
| Age | -0.001 | 0.001 | 0.566 | 0.000 | 0.001 | 0.86 |

HCV: Hepatitis C virus; HIV: human immunodeficiency virus; HD: Healthy donor; MEU: Multiple exposed but uninfected with HCV or HIV; SE: standard error. * HD at T=1 and T=2 are not the same individuals
