# Supplementary material for: HCV monoinfection and HIV/HCV coinfection enhance T-cell immune senescence in injecting drug users early during infection
Source: Immun Ageing. 2016 Mar 31;13:10. doi: 10.1186/s12979-016-0065-0 (PMC4815107; doi:10.1186/s12979-016-0065-0)
Supplement: Additional file 2: — Flowcytometric analysis of telomere length. Example of flowcytometric analysis of telomere length by flow-FISH, where calf thymocytes (red) can be distinguished from lymphocytes (blue), not only by forward and sideward scatter (left panel) but also by the lack of CD3 expression (right panel) (A&B). The cells were either hybridized to the peptide nucleic acid (PNA) probe (D) or underwent the same experimental conditions without the PNA probe (c) to account for the level of autofluorescence. (PDF 216 kb) [file 12979_2016_65_MOESM2_ESM.pdf]

Supplemental figure

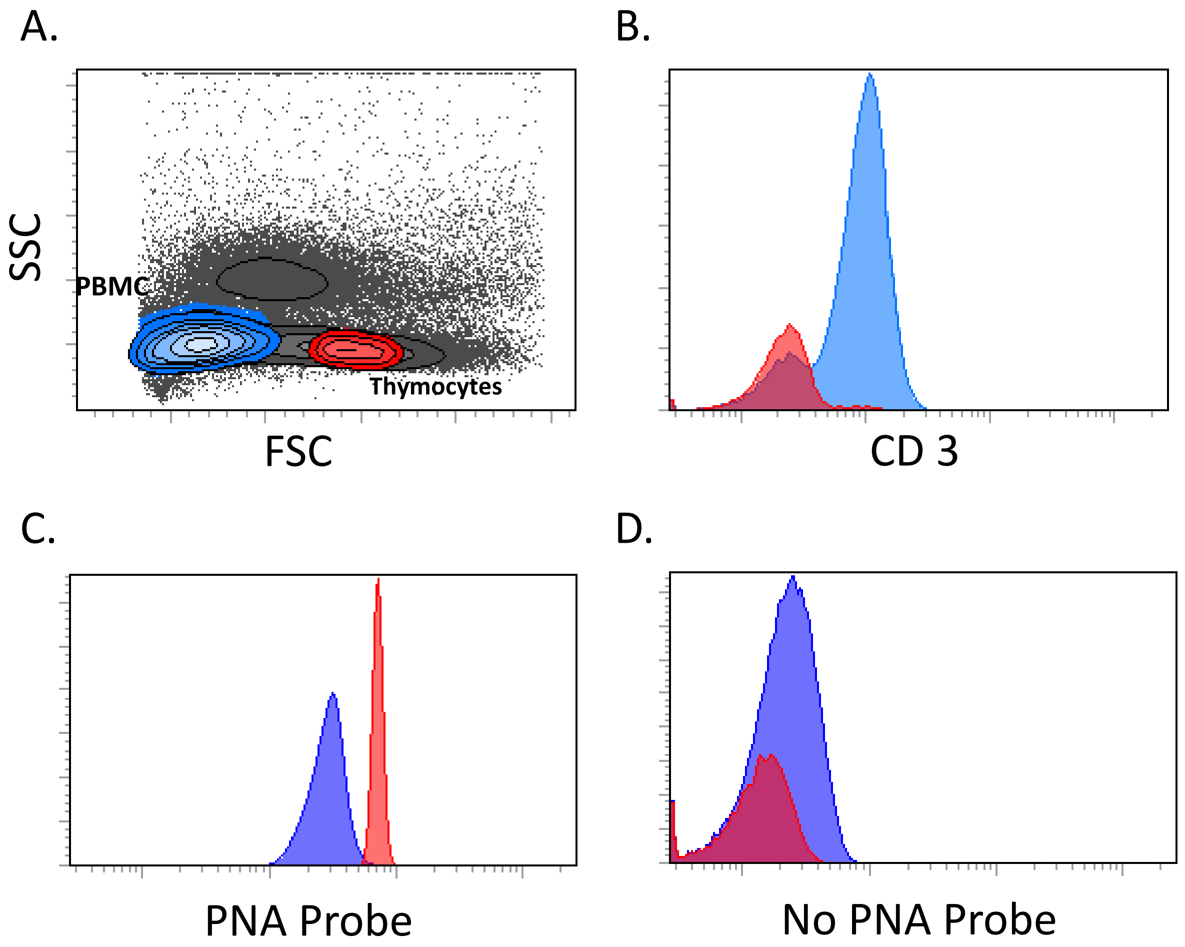

Flowcytometric analysis of telomere length.

Example of flowcytometric analysis of telomere length by flow-FISH, where calf thymocytes (red) can be distinguished from lymphocytes (blue), not only by forward and sideward scatter (left panel) but also by the lack of CD3 expression (right panel) (A&B). The cells were either hybridized to the peptide nucleic acid (PNA) probe (D) or underwent the same experimental conditions without the PNA probe (c) to account for the level of autofluorescence.
